# Methylation Status of the Telomerase Reverse Transcriptase Promoter in Parotid Tumours and Adjacent Parotid Gland Tissue: A Pilot Study on the Implications for Recurrence and Development of Malignancy

**DOI:** 10.3390/curroncol32060312

**Published:** 2025-05-28

**Authors:** António Paiva-Correia, Joana Apolónio, Alfons Nadal, José Ricardo Brandão, Nádia Silva, Bianca Machado, Ivan Archilla, Pedro Castelo-Branco, Henrik Hellquist

**Affiliations:** 1 Faculty of Medicine and Biomedical Sciences (FMCB), University of Algarve, Gambelas Campus, Bld. 2, 8005-139 Faro, Portugal; joana.apolonio@abcmedicalg.pt (J.A.); nsilva@ualg.pt (N.S.); henrikhellquist@pm.me (H.H.); 2 Algarve Biomedical Center Research Institute (ABC-RI), University of Algarve, Gambelas Campus, 8005-139 Faro, Portugal; 3 Cellular Pathology Department, Manchester University NHS Foundation Trust, Wythenshawe Hospital, Manchester M23 9LT, UK; 4 Department of Pathology, Hospital Clinic, 08036 Barcelona, Spain; 5 Department of Basic Clinical Practice, School of Medicine, University of Barcelona, 08007 Barcelona, Spain; 6 Unidade local de Saude de Santo Antonio, 4099-001 Porto, Portugal; 7 Unilabs, Avenida de França, 434, 4050-277 Porto, Portugal; 8 Instituto Português de Oncologia do Porto (IPO-Porto), Anatomia Patologica, 4100-321 Porto, Portugal; u10803@chporto.min-saude.pt; 9 Champalimaud Research Program, Champalimaud Centre for the Unknown, 1400-038 Lisbon, Portugal; 10 Department of Cellular Pathology, Northern Lincolnshire and Goole NHS Foundation Trust, Lincoln 999039, UK

**Keywords:** salivary gland neoplasms, cancer, pleomorphic adenoma, h*TERT*, THOR, methylation, biomarkers

## Abstract

Background/Objectives: The methylation of the hypermethylated oncological region (THOR) of human telomerase reverse transcriptase (hTERT) may forecast tumour aggressiveness. This pilot study aimed to evaluate THOR methylation as a potential biomarker for recurrence/malignant transformation in salivary gland pleomorphic adenomas (PA). Methods: THOR methylation was assessed by quantitative pyrosequencing in 96 parotid tissue samples (benign and malignant), including non-neoplastic parotid tissue, PA, recurrent PA (rPA), and carcinomas, along with their adjacent tissues. TERT promoter mutations (TPMs) were analysed by Sanger sequencing. Results: THOR methylation significantly differed across the seven groups. Malignant tissues showed higher THOR methylation than non-neoplastic tissues, whereas benign tumours showed no significant difference from non-neoplastic tissue. THOR methylation in rPA was closer to carcinoma than to normal tissue, similar in rPA and tissues adjacent to rPA, and higher in tissues adjacent to carcinomas than in non-neoplastic tissues. A subset of PA-adjacent tissues showed epigenetic alterations, suggesting an increased risk of recurrence or malignant transformation (5–15%). No TPMs were detected. Conclusions: THOR methylation may add information to differentiate normal from carcinogenic tissues and, as such, may be included in a biomarkers panel. Epigenetic alterations in PA-adjacent tissues with normal histology highlight the need for improved diagnostic markers.

## 1. Introduction

Telomeres are repetitive nucleoprotein structures that protect chromosomal ends and play important roles in cell division, genome stability, and cancer prevention [1,2]. The length of the telomeres shortens after each replicative cycle, with implications on cell death and ageing/senescence processes. To achieve replicative immortality, about 90% of human cancers maintain the length of telomeres by reactivating telomerase, which is driven by the transcriptional upregulation of human telomerase reverse transcriptase (h*TERT*), through re-expression of the catalytic subunit h*TERT* [3].

Contrary to gene mutations, alterations of the epigenetic mechanisms (“epimutations”) are reversible and, thereby, can be appealing as therapeutic targets. Telomere maintenance is one of the most important hallmarks of cancer, and, moreover, replicative immortality is an attribute of cancer cells governed in most cases by telomere maintenance [4]. In this context, we identified a specific methylation signature in the h*TERT* gene, named TERT Hypermethylated Oncological Region—THOR [5]. We have also described THOR as a cancer-associated epigenetic mechanism of h*TERT* upregulation, with unmethylated THOR repressing TERT promoter activity regardless of TERT promoter mutations (TPMs) status [6,7,8]. The hypermethylation of THOR counteracts this repressive function, thus proposing that THOR hypermethylation is a prevalent telomerase-activating mechanism in cancer that can act independently of or in conjunction with TPMs. This will further support the utility of THOR status as a prognostic biomarker [9]. Also, the hypermethylation of THOR predicts recurrence and survival in pancreatic cancer, predicts biochemical relapse in prostate cancer, and is a promising biomarker in breast cancer [6,7,8]. Telomerase is, thus, a crucial enzyme for the limitless self-renewal of cells and is expressed in most tumours, allowing for cancers to progress and recur. Even though the recurrence of PA is also related to the applied treatment, the extent of parotidectomy, histological features of PA, non-radical excision, or tumour spillage on the surgical side [10,11], recent studies have indicated the role of TERT mutations as a very strong predictor of tumour recurrence in hepatocellular carcinoma [12]. THOR methylation was not studied, but TERT promoter mutations were, and it does merit publication in the literature of the ever-opening field of pathology [13].

Amongst benign salivary neoplasms, pleomorphic adenoma (PA) is standing out as having a relatively high risk of recurrence and development into malignancy (5–15%) [14]. THOR methylation status has already been demonstrated to have diagnostic and prognostic utility in several tumours of other organs, and we hypothesize that THOR methylation may be of interest not only in cancers but also in pleomorphic adenoma and in the parotid tissue adjacent to PA. We believe that one key event to recurrence and malignancy in PAs may be THOR methylation-based epigenetic mechanisms controlling PA tumorigenesis and biological behaviour.

However, a systematic evaluation of THOR methylation status on these tissues has not been performed so far, and no clinical, histological, immunohistochemical, or molecular criteria for the described aggressive behaviour have been reported in the literature.

In this study, we investigated THOR methylation status and TPM analysis in parotid PAs and salivary carcinomas and compared normal parotid gland tissue, i.e., parotid tissue from glands without any neoplastic disease (tissue excised due to cyst, calculus, etc.), adjacent parotid tissue taken from glands containing PA and recurrent PA, as well as tissue adjacent to carcinomas.

The main objective of this pilot study was to evaluate THOR methylation status as a predictive biomarker of parotid salivary gland PA recurrence and/or malignant transformation. With this, we expect to provide information to improve the development of screening and early diagnostic tools for PA in particular and open new possibilities regarding targeted treatment.

## 2. Materials and Methods

### 2.1. Materials

In this study, parotid tissue samples were collected from archival routine material, and, hence, the study materials consisted of formalin fixed tissue processed and embedded in paraffin blocks (FFPE tissue), as performed in a routine histopathology laboratory. The inclusion criteria for sample collection were as follows: (i) benign parotid tumours, including PA and parotid recurrent PA; (ii) primary parotid gland carcinoma samples only, including mucoepidermoid carcinoma (MEC), acinic cell carcinoma (ACC), adenoid cystic carcinoma (AdCC), basal cell adenocarcinoma (BCA), carcinoma ex pleomorphic adenoma (CxPA), and epithelial-myoepithelial carcinoma (EMC) from the archives of four institutions (Unidade Local de Saúde de Santo António; IPO Porto; Hospital Clinic Barcelona; Northern Lincolnshire and Goole NHS Foundation Trust); (iii) histological samples with an appropriate amount of tumour tissue and adjacent non-tumoral tissue for THOR methylation analysis; (iv) samples of tissues from non-neoplastic parotid glands; (v) samples from patients with complete follow-up clinical records; (vi) samples from patients with age between 15 and 79 years.

The exclusion criteria for sample collection were as follows: (i) samples with an insufficient amount of tissue for analysis; (ii) samples with evidence of high levels of inflammation (confirmed by the presence of a prominent inflammatory infiltrate under the microscope); (iii) samples from patients less than 15 and more than 79 years of age; (iv) samples from metastatic tumours and lymphomas; (v) benign and malignant parotid gland samples from fine-needle aspirates (FNAs), cytology in general, and biopsies.

The respective age, gender, treatment procedure(s), and follow-up of the patients from whom the samples were collected were noted, and the cases were randomly coded and incorporated into seven different validation and discovery groups of samples.

From the initial 122 parotid tissues collected as samples, 5 samples of patients over 79 years old were excluded, as comorbidities are associated with higher methylation of promoter genes (all patients over 79 years old in our samples died of other causes) [15]. After excluding 8 samples with insufficient DNA content for THOR methylation analysis, 4 samples with “no amplification data” results, and 9 other potentially inadequate samples (with potential sampling errors found after a detailed second review of H&E slides and paraffin blocks), 96 final valid samples were included for analysis of THOR methylation status. These 96 samples included both benign (tissue from PA and tissue adjacent to PA) and malignant (MEC, ACC, AdCC, CxPA, and EMC) tumours and also normal parotid tissue and were organized in seven groups (Table 1): normal parotid tissue from non-neoplastic glands (group 1, *n* = 9); tissue from PA (group 2, *n* = 22); parotid tissue adjacent to PA (group 3, *n* = 17); tissue from recurrent PA (rPA, group 4, *n* = 12); parotid tissue adjacent to rPA (group 5, *n* = 9); tissue from parotid carcinoma, including 3 MEC, 3 ACC, 2 AdCC, 3 CxPA, and 3 EMC (group 6, *n* = 14); and parotid tissue adjacent to parotid carcinoma (group 7, *n* = 13). Tumour samples and glandular tissues adjacent to those tumours were paired samples from the same patients.

### 2.2. Methods

#### 2.2.1. Tissue Extraction

Tissues were carefully extracted from the FFPE blocks by using a 2 mm punch (standard sterile disposable skin punch and a separate punch for every single sample extraction). Punch extractions were performed after correlation between the FFPE block and marked areas on the corresponding H&E-stained slide (performed by two histopathologists). The tissues were stored in sterile tubes.

#### 2.2.2. DNA Isolation

The tissues obtained from the FFPE blocks were dewaxed with xylene, and then the genomic DNA was extracted from the tissues using the DNeasy Blood and Tissue Kit (Qiagen, Hilden, Germany) according to the manufacturer’s protocol. The tumour area and the tumour-adjacent parotid tissue of each FFPE tissue block were carefully selected to prevent contamination. After DNA extraction, DNA concentration was measured using the Nanodrop 2000 system (Thermo Scientific, Waltham, MA, USA). All samples were kept at −20 °C before THOR methylation analysis.

#### 2.2.3. THOR Methylation Analysis

The analysis of THOR methylation was performed through quantitative sodium bisulfite pyrosequencing, as previously described [5], at the Genomic Core Facility of IBIMA (Biomedical Institute of Malaga, Malaga, Spain). Briefly, for quantitative sodium bisulfite pyrosequencing analysis, 500 ng of genomic DNA was treated with sodium bisulfite using EZ DNA Methylation Kit (Zymoresearch, Tustin, CA, USA, D5001) according to the manufacturer’s procedure. The region of interest was then amplified by PCR and followed by pyrosequencing, which was carried out using PyroMark Q24 (Qiagen) according to the manufacturer’s protocol (Pyro-Gold reagents). Targeted assays were designed using the PyroMark Assay Design Software 1.0 (Qiagen). Forward ATGATGTGGAGGTTTTGGGAATAG, reverse CCCAACCTAAAAACAACCCTAAAT, and sequencing GGAGGTTTTGGGAATAG primers were used for PCR and pyrosequencing. The assay target region within THOR was 36 bp in length, comprising 5 CpG sites (chr5:1295586, chr5:1295590, chr5:1295593, chr5:1295605, chr5:1295618, GRCh37/hg19 genome assembly). The calculation of the percentage of THOR methylation was performed as the average value of these 5 CpG sites.

#### 2.2.4. TERT Promoter Mutations (TPMs) Analysis

After the initial THOR methylation analysis, 30 samples were sent to Eurofins Genomics, Germany, for Sanger sequencing to identify specific TERT promoter mutations, as previously described [8,9,16]. These samples included 6 tissue samples from PA, 6 tissue samples from rPA, 6 tissue samples adjacent to PA, 6 tissue samples adjacent to rPA, and 6 tissue samples from carcinoma (1 MEC, 2 AdCC, 2 EMC, and 1 CxPA).

Sanger sequencing of PCR products was used to identify specific h*TERT* promoter mutations (1,295,250 G>A and 1,295,228 G>A, C>T on opposite strand) in tissues and controls (MDA-MB-231 and MCF-7). MDA-MB-231 and MCF-7 breast cancer cell lines were used as positive (harbours C228T mutation) and negative controls, respectively.

Following DNA extraction, a 100-base pair (bp) PCR amplicon encompassing the proximal h*TERT* promoter was amplified using primers complementary to genomic DNA with added sequencing tag overhangs:

5′-ACACTGACGACATGGTTCTACA-GGCCGCGGAAAGGAAGGGG (forward); 5′-TACGGTAGCAGAGACTTGGTCT-CGCCTCCTCCGCGCGGAC (reverse).

The PCR was run in 20 μL reactions composed of 10 μL of HotStarTaq *Plus* Master Mix DNA polymerase (Qiagen, 203643), 0.5 μL of each primer (10 μM), 1 μL of glycerol, 7 μL of H_2_O, and 1 μL of genomic DNA (50 ng). PCR conditions were the following: 95 °C for 5 min, followed by 40 cycles of denaturation at 95 °C for 30 s, annealing at 64 °C for 1 min, extension at 72 °C for 45 s, and one cycle for a final extension at 72 °C for 7 min. After PCR amplification, 4 μL of each product was run on a gel to confirm if the product was successfully amplified. The resulting PCR product was purified using the QIAquick PCR Purification Kit (Qiagen, 28106), and 50 ng of DNA was sequenced both in the forward and reverse directions using 5′-ACACTGACGACATGGTTCTACA and 5′-TACGGTAGCAGAGACTTGGTCT sequencing primers, respectively. Mutations were recognized on sequencing electropherograms.

#### 2.2.5. Statistical Approach, Programme, and Sample Size Calculations

From the 114 initial samples analysed for THOR methylation, 110 (the results of 4 cases came back with “no amplification data”) were statistically analysed using GraphPad Prism 8.0 software; a total of 96 samples were considered valid for statistical analysis after the exclusion of 14 samples (reasons detailed in the Material subsection).

Discrete variables were summarized through counts (percentages), and continuous variables were summarized through the median value (interquartile range [Q1, Q3]). To assess the difference in THOR methylation between non-neoplastic (“normal” control tissue) and tumour tissues, a two-tailed Mann–Whitney U test was used. To test the difference in THOR between salivary gland tumours and histopathological groups, the Kruskal–Wallis test was used. A *p*-value below 0.05 was considered statistically significant.

## 3. Results

### 3.1. Characteristics of the Population

Our obtained samples valid for statistical analysis, 96 in total, were derived from 57 individuals with a diagnosis of non-neoplastic or neoplastic parotid disease and organized in seven groups. The characteristics of the patients are summarized in Table 1, Table 2, Table 3, Table 4 and Table 5. Of the 57 patients, 35 were female and 26 were more than 50 years old with a median age of 47 (35.5-Q1; 61.5-Q3) years. Superficial parotidectomy (SPE) was performed in 65% of the patients; 17.5% of these patients received radiotherapy after surgery (tissue collection). The median follow-up was 131 months. The studied groups comprised 9 to 22 samples, as illustrated in Table 1. From the overall group, 42 patients showed no signs of disease at their last follow-up visit whilst 8 patients with PA had recurrent disease (2 of them twice). Six patients were lost to follow-up and seven patients died of other causes (Table 2, Table 3, Table 4 and Table 5).

### 3.2. THOR Methylation

**Figure 1:**
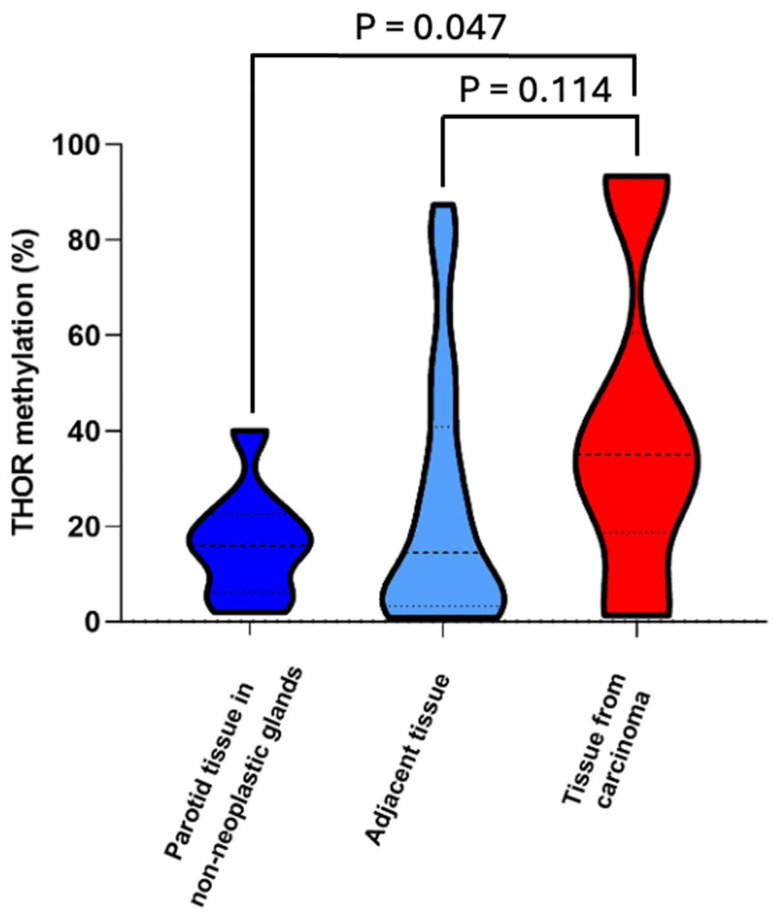
THOR methylation in parotid tissue (non-neoplastic glands), tissue from all adjacent parotid gland tissues, and tissue from parotid gland carcinoma. Dotes lines correspond to Q1, median, and Q3.

The overall THOR methylation status for the seven different groups showed a marked difference between several groups, particularly between the non-neoplastic parotid tissue compared with carcinomas (40.00%), PAs (27.98%), rPA (37.17%), and parotid tissue adjacent to carcinomas (21.71%) (Supplementary Figure S1; Table 2, Table 3, Table 4 and Table 5). In particular, the comparison between non-neoplastic (“normal” control tissue) and tissues from salivary gland carcinoma (Figure 1) demonstrated that carcinomas show considerably higher THOR methylation (14.83% vs. 40.00%; *p* = 0.047).

Six of the nine investigated samples of parotid tissue from non-neoplastic glands (Group 1) had a THOR methylation < 20%, and no difference was found between THOR methylation in recurrent PA and respective adjacent tissues (*p* = 0.844).

**Figure 2:**
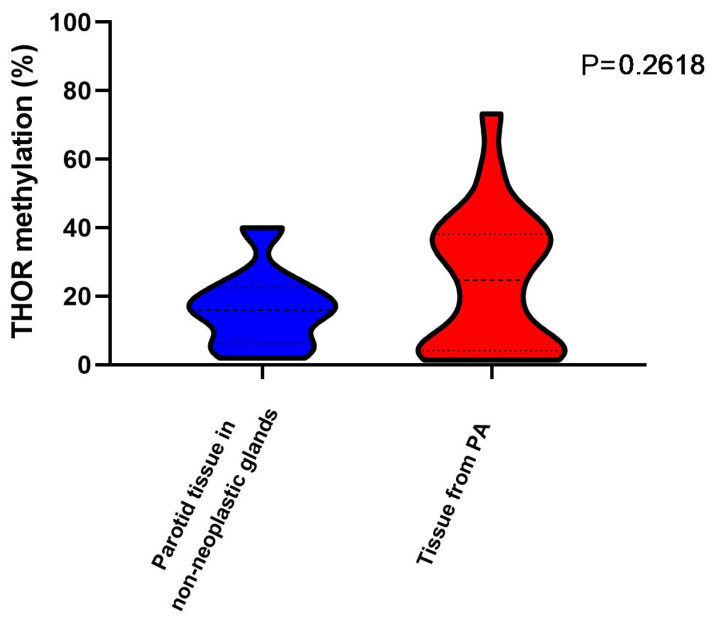
THOR methylation in parotid tissue (non-neoplastic tissue) and tissue from pleomorphic adenoma (PA). Dotes lines correspond to Q1, median, and Q3.

Among PA, our analysis found epigenetic heterogeneity in 20% to 30% of the cases (Figure 2), with some tissues showing THOR epigenetic characteristics closer to carcinomas.

**Figure 3:**
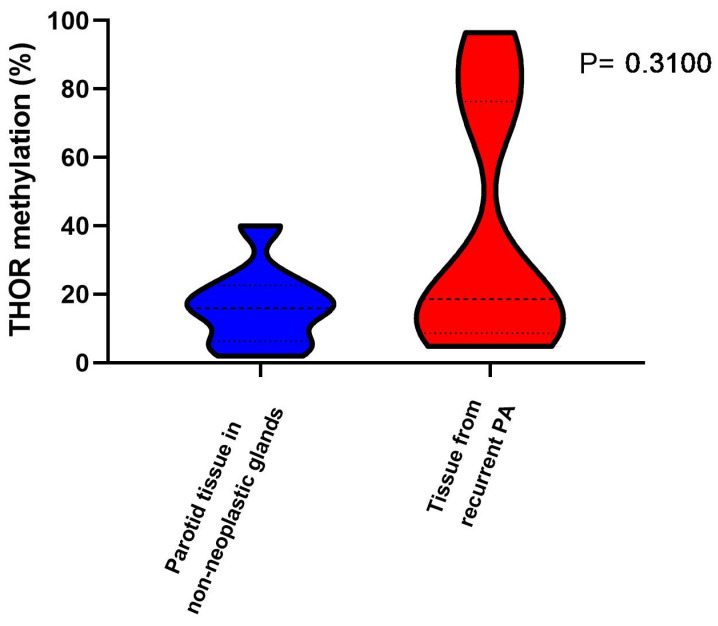
THOR methylation in parotid tissue (non-neoplastic tissue) and tissue from recurrent pleomorphic adenoma (PA). Dotes lines correspond to Q1, median, and Q3.

In addition, the 12 rPAs showed an average THOR methylation closer to carcinoma than to “normal” tissue (Figure 3). Regarding the comparison of THOR methylation in non-neoplastic benign recurrent tumour tissues and tissues adjacent to recurrent tumour, no significant differences were found (*p* = 0.843).

Concerning all the groups of adjacent tissues, even though average THOR methylation was closer to normal, a subgroup presented epigenetic alterations evidenced by THOR hypermethylation when compared with non-neoplastic tissues (Figure 2).

Based on previous studies in tumours of other organs, 20% was considered as the threshold above which THOR methylation percentage could be considered as hypermethylation [8,9]. When all cases (Table 1) were grouped into non-neoplastic samples (groups 1, 2, 4, and 6), hypermethylation (>20%) occurred in 15 out of 48 cases (31%), suggesting that some of these samples might be in the process of carcinogenic transformation. In fact, all the samples that presented hypermethylation in this group belong to tissues adjacent to PA, rPA, and carcinomas, and, therefore, what are considered adjacent normal tissues might not be at least at an epigenetic level. In 17 out 44 PAs (groups 3 and 5), 50% were hypermethylated but 11 of 14 carcinomas were hypermethylated (79%) (Table 6). Comparing non-malignant samples (non-neoplastic + PAs and rPAs) versus carcinomas, differences in distribution remained statistically significant: 35 out of 82 among non-carcinoma samples versus 11 out of 14 carcinomas were hypermethylated. Similarly, 38% of non-neoplastic samples were hypermethylated versus 58% of neoplastic samples (PAs + rPAs + carcinomas) (Table 6).

### 3.3. TERT Promoter Mutations (TPMs) Analysis Results

Regarding the TPM analysis by Sanger sequencing to identify the two hotspot positions (−124 G>A (G228A) and −146 G>A (G250A), C>T on opposite strand), no TPM mutations (G228A and G250A) were found in any of our 30 samples. As expected, no TPM were identified in MCF-7 (C-) negative controls, and the TPM G228A was present in MDA-MB-231 (C+).

## 4. Discussion

In this study, we evaluated THOR methylation status and TPM in parotid PAs and SGT and compared the results with those obtained for “normal” parotid gland tissue, including parotid tissue from glands without any neoplastic disease, tissue adjacent to PA and to carcinomas, and rPA tissues. Our results add valuable information to the very limited existing knowledge on the epigenetics of SGT [17,18,19,20].

Data from previous studies in tumours of other organs show that normal tissues have a basal THOR methylation of around 15% to 20% [8,9]. In this context, 20% has been considered as a threshold above which THOR methylation percentage can be considered as hypermethylation [8,9]. THOR hypermethylation is not observed in normal tissues with high hTERT expression and a high cellular division rate (as T lymphocytes and germinative cells). In fact, THOR hypermethylation is a feature specific to cancer; non-cancer cells activate or repress h*TERT* via other mechanisms.

Our results showed that, except for non-neoplastic tissues, all the other samples showed hypermethylation, apart from a few outliers. We also verified that tissues from carcinoma samples (group 7) had a median THOR methylation higher than that observed in all the other groups. Overall, the differences observed between non-neoplastic tissues and SGT showed that SGT is epigenetically regulated, at least by THOR methylation. This means that THOR methylation provides relevant information to differentiate between “normal”/benign tissues and carcinogenic tissues, suggesting that we found a probable biomarker of malignancy in SGT.

Of note, despite looking histologically normal, a subgroup of adjacent tissues showed epigenetic alterations evidenced by THOR hypermethylation, i.e., the tissues were not normal as they already presented THOR patterns of malignancy. The results of this subgroup suggest that THOR methylation may forecast the aggressive behaviour of SGT and adjacent tissues, as previously demonstrated by members of our group for tumours of other organs, such as the breast [8]. It is possible that these alterations are occurring at an epigenetic level prior to histopathological observations and may drive the transformation of tissues adjacent to the observed lesion. Overall, histologically normal tissues may not be normal at an epigenetic level (they may be pre-malignant).

The subgroup of PA (20% to 30% of the PA group) with THOR patterns closer to malignancy evidenced PA epigenetic heterogeneity and may potentially recur or develop malignant transformation. This trend agrees with our previous review on this matter, in which we stated that 5% to 15% of PA recur and/or develop malignant transformation [14].

Our findings indicate that THOR methylation levels in recurrent PA tissues are closer to those observed in carcinomas than in benign PA, suggesting that these tumours may possess an intrinsic predisposition to recurrence or malignant transformation. Moreover, hypermethylation detected in histologically normal adjacent tissues highlights the possibility of a “field cancerization” effect, where epigenetic changes precede histological malignancy. The recurrence of PA has been associated with surgical factors such as tumour spillage, incomplete excision, or capsule rupture, but emerging molecular insights suggest that epigenetic alterations may also contribute to its pathogenesis. These results support the need for long-term monitoring of patients with PA, particularly those with recurrent disease, and suggest that molecular profiling, including THOR methylation assessment, could aid in identifying cases at higher risk of recurrence or malignancy.

Although THOR methylation status can also be assessed in FNA following DNA extraction and the same analytical protocol, this study included only excisional specimens to increase the availability of representative tissue and with sufficient DNA for methylation analysis. Therefore, FNA and biopsy samples were excluded, as they typically provide more limited tissue for analysis. Nonetheless, given the differences observed in THOR methylation status among carcinomas, PA, rPA, and normal tissues, this molecular test might contribute to establish a diagnosis using FNA biopsy, as already demonstrated in other organs [21]. In addition, the absence of TPM mutations confirmed that these tumours may regulate the expression of telomerase mainly epigenetically rather than genetically. However, transactivation effect from TERT regulation (i.e., abnormalities in transcription factors) cannot be excluded. Still, our results agree with those reported for benign tumours or malignant SGT [22,23]. The obtained results are closer to data obtained from prostate and breast tumours than to data from brain and urinary bladder tumours. This may be correlated with the similarities, in terms of histogenesis and origin cell type, between SG and prostate and breast cells, which are not observed between SG and brain and urinary bladder cells.

This study presents some limitations that deserve to be discussed. First, the sample size is limited and, as such, for broader conclusions, studies with larger samples are warranted. However, considering that this a pilot study, the results are informative and support future studies in larger groups of samples. Second, the group of samples was heterogeneous regarding the type of cancer and the origin of samples. Third, the design of our study did not enable the comparison of THOR methylation in rPA and PA tissues of the same patient, as at the start of the study, we could not identify the tissues that recurred nor those that came to recur later. Ideally, the study sample should include patients with primary PA and rPA previously operated on in the same centre, and THOR methylation should be assessed in the same patient in primary and recurrent samples of PA and compared with patients with primary PA who did not recur within 5 years after surgery.

## 5. Conclusions

We show that SGT is epigenetically regulated by THOR methylation, which opens possibilities regarding the clinical management of patients, mainly in terms of target therapy for these patients. We believe that histone deacetylase (HDAC) inhibitors or small-molecule drugs targeting the telomerase pathway may help modulate THOR methylation and its downstream effects [24]. Also, THOR methylation proves to be of value in differentiating non-neoplastic (group 1, “normal” control tissue) from carcinoma, and, potentially, from recurrent PA tissue and parotid tissue adjacent to carcinoma and tissues adjacent to carcinoma and to PA. As such, THOR methylation may be included in a biomarkers panel, which can be used for malignancy diagnosis or confirmation.

To further clarify and generalize our findings, future studies with larger samples and longer follow-up periods, and including a THOR comparative analysis of PA, rPA, and carcinoma ex-PA (CxPA) tissues of the same patient, are warranted.

## Figures and Tables

**Table 1:** Demographic and clinical characteristics of the study population.

Characteristics	Study Population (*n* = 57)
Gender	
Female	35 (61%)
Male	22 (39%)
Group	
Normal tissue from non-neoplastic parotid glands	9 (9%)
2. Tissue from PA	22 (23%)
3. Parotid tissue adjacent to pleomorphic adenoma (PA)	17 (18%)
4. Tissue from recurrent PA *	12 (13%)
5. Parotid tissue adjacent to rPA *	9 (9%)
6. Tissue from carcinoma **	14 (15%)
7. Parotid tissue adjacent to salivary gland carcinoma	13 (14%)
Age, years	47.0 (35.5, 61.5)
Follow-up, months	131 (51.2, 159)
Treatment	
SPE	37 (65%)
PR	12 (21%)
Parotidectomy	7 (12%)
RT	10 (18%)
ENU	1 (2%)

Results are presented as median (Q1, Q3) for non-normal continuous variables and *n* (%) for categorical variables. * rPA recurrent PA but not recurrent to any PA investigated in this study, hence separate and unrelated cases. ** 3 mucoepidermoid carcinomas (MEC), 3 acinic cell carcinomas (ACC), 2 adenoid cystic carcinomas (AdCC), 3 carcinoma ex pleomorphic adenomas (CxPA), 3 epithelial-myoepithelial carcinomas (EMC); ENU: enucleation; PR: partial resection; RT: radiotherapy; SPE: superficial parotidectomy.

**Table 2:** THOR methylation status in parotid tissue from non-neoplastic glands (*n* = 9).

Sample	Diagnosis	Age * (Years)	Sex * (F/M)	Treatment *	Follow-Up (Months) *	THOR Methylation (%)
49	Cyst	51	F	SPE	60, NED	20.66
80	Infl	44	F	SPE	60, NED	1.91
83	Cyst	71	M	SPE	36, NED	7.96
84	Cyst	65	M	SPE	84, NED	4.58
85	Cyst	16	F	SPE	82, NED	1.94
86	Cyst	64	F	SPE	36, NED	24.53
88	Cyst	47	M	SPE	60, NED	40.04
110	Cyst	64	M	SPE	66, NED	17.44
111	Cyst	53	M	PR	96, NED	14.44

F: female; Infl: inflammation; M: male; NED: no evidence of disease; PR: partial resection; SPE: superficial parotidectomy. * of the patients of origin.

**Table 3:** THOR methylation in adjacent tissue to pleomorphic adenoma (*n* = 17) and pleomorphic adenoma (*n* = 22).

Adjacent Tissue Sample Code	THOR Methylation (%)	PA Tissue Sample Code	THOR Methylation (%)	Age * (Years)	Sex * (F/M)	Treatment *	Follow-Up (Months) *
No AT	-	21	43.56	15	F	SPE	240, NED
22	51.45	23	33.10	61	F	SPE	240, NED
24	83.73	25	36.32	33	F	SPE, RT	96, NED
26	24.60	27	56.98	17	M	SPE	250, NED
28	8.04	29	45.86	46	F	SPE	200, NED
30	87.43	31	30.22	34	F	SPE	240, NED
32	3.77	33	2.95	58	M	SPE	70, DOC
34	0.83	35	1.34	58	M	SPE	90, NED
36	10.67	37	18.41	56	F	SPE	220, NED
No AT	-	38	73.27	20	M	SPE	250, NED
No AT	-	39	3.65	25	F	SPE	250, NED
40	1.54	41	4.66	28	M	SPE	L
44	2.89	45	8.04	38	M	SPE	250, NED
No AT	-	46	39.93	47	M	SPE	130, NED
53	2.02	52	2.93	38	F	SPE	132, NED
55	55.81	54	27.98	36	M	PR	60, NED
69	16.48	70	21.37	38	F	SPE	37, NED
No AT	-	72	37.48	50	F	SPE	37, NED
75	23.79	76	4.29	48	F	SPE	21, NED
No AT	-	82	1.61	41	F	ENU	132, NED
89	12.07	90	36.47	39	M	SPE	192, NED
107	1.85	108	**	29	F	ENU	228, NED
120	6.59	121	13.27	24	F	SPE	252, NED

* of the patients of origin. ** There are no THOR methylation data for this sample, poor quality DNA, no amplification; this sample was not considered for statistical analysis and, as such, is not included in Table 1. DOC: died from other causes; ENU: enucleation; L: lost for follow-up; F: female; M: male; NED: no evidence of disease; No AT: no adjacent tissue; PR: partial resection; RT: radiotherapy given 6 months after surgery; SPE: superficial parotidectomy.

**Table 4:** THOR methylation in rPA (*n* =12) and its tissues adjacent to (*n* = 9).

Adjacent Tissue Sample Code	THOR Methylation (%)	rPA Tissue Sample Code	THOR Methylation (%)	Age * (Years)	Sex * (F/M)	Treatment *	Follow-Up * (Months)
57	80.84	56	88.99	78	F	SPE	84, NED
91	5.81	92	4.77	32	F	PR	192, NED
93	19.36	94	16.88	41	F	PE	240, NED
97	77.85	98	19.74	59	F	SPE	216, NED
101	42.19	102	**	36	F	PR	144, NED
103	15.79	104	5.08	33	F	SPE	36, R, L
105	22.10	106	17.26	64	F	PR	36, AWD
113	6.34	114	78.33	55	F	PR	12, R, L
118	4.14	119	7.96	26	F	PR	228, NED
No AT	-	81	96.58	51	F	P	30, R
No AT	-	112	29.33	41	M	PR	24, R, L
No AT	-	117	70.37	35	F	PR	156, NED
No AT	-	122	10.75	56	F	PR	12, R, L

* of the patients of origin. ** There is no THOR methylation data item for this sample, poor quality DNA, no amplification; this sample was not considered for statistical analysis and, as such, is not included in Table 1. AWD: alive with disease; L: lost for follow-up; F: female; M: male; NED: no evidence of disease; No AT: No adjacent tissue; PE: parotidectomy; PR: partial resection; rPA: recurrent pleomorphic adenoma; R: recurrence; SPE: superficial parotidectomy.

**Table 5:** THOR methylation in tissues adjacent to salivary gland carcinomas (*n* = 13) and salivary gland carcinomas (*n* = 14).

Adjacent Tissue Sample Code	THOR Methylation (%)	Carcinoma Tissue Sample Code	THOR Methylation (%)	Age * (Years)	Sex * (F/M)	Treatment *	Follow-Up (Months)
No AT	-	2 (ACC)	38.54	15	F	PR	140, NED
5	67.64	6 (MEC)	2.77	53	F	SPE, RT	127, NED
7	1.74	8 (AdCC)	1.29	44	M	PR	84, NED
9	1.57	10 (EMC)	1.29	64	M	PE	126, NED
11	51.84	12 (ACC)	25.57	75	M	PE, RT	130, DOC, NED
13	33.36	14 (EMC)	**	76	M	SPE	90, DOC, NED
16	30.91	17 (MEC)	50.35	63	F	SPE	116, NED
No AT	-	18 (ACC)	42.07	41	M	SPE	134, NED
19	14.44	20 (CxPA)	91.29	76	M	PR	L
No AT	-	47 (AdCC)	91.70	41	F	SPE, RT	144, NED
51	3.23	50 (CxPA)	23.91	70	F	SPE	24, NED
59	4.55	60 (ACC)	**	75	F	PE, RT	109, NED
61	40.83	62 (EMC)	36.51	43	F	PR, RT	137, NED
63	1.48	64 (EMC)	93.44	61	F	PE, RT	136, NED
65	29.01	66 (BCA)	**	68	F	PE	24, DOC
No AT	-	68 (MEC)	33.56	72	M	SPE, RT	56, NED
95	1.63	96 (CxPA)	27.68	62	M	PR	168, NED, DOC

* of the patients of origin. ** There are no THOR methylation data for this sample, poor quality DNA, no amplification; this sample was not considered for statistical analysis and, as such, is not included in Table 1. ACC: acinic cell carcinoma; AdCC: adenoid cystic carcinoma; BCA: basal cell adenocarcinoma; CxPA: carcinoma ex pleomorphic adenoma; DOC: died from other causes; EMC: epithelial-myoepithelial carcinoma; F: female; L: lost for follow-up; M: male; MEC: mucoepidermoid carcinoma; NED: no evidence of disease; PE: parotidectomy; RT: radiotherapy; SPE: superficial parotidectomy. * Due to low amount of DNA, these two samples were selected for future EPIC array methylation analysis only; these samples were not considered for statistical analysis and, as such, are not included in Table 1.

**Table 6:** Chi-squared test of the distribution of hypermethylation positivity (>20%) between the different categories of samples.

	Non-Neoplastic	PAs and rPAs	Carcinomas *
>20%	18	17	11
<20%	30	17	3
*p* = 0.025			
	**Non-neoplastic + PAs and rPAs**		Carcinomas *
>20%	35		11
<20%	47		3
*p* = 0.013			
	**Non-neoplastic**		Neoplastic *
>20%	18		28
<20%	30		20
*p* = 0.041			

PAs: pleomorphic adenomas; rPAs: recurrent pleomorphic adenomas. * 3 acinic cell carcinomas (ACC), 2 adenoid cystic carcinomas (AdCC), 3 carcinoma ex pleomorphic adenomas (CxPA), 4 epithelial-myoepithelial carcinomas (EMC), and 2 mucoepidermoid carcinomas (MEC).

## Data Availability

The raw data supporting the conclusions of this article will be made available by the authors on request.

## Supplementary Materials

The following supporting information can be downloaded at: https://www.mdpi.com/article/10.3390/curroncol32060312/s1, Figure S1: THOR methylation in the seven groups.

## List of Abbreviations

CxPA	carcinoma ex-pleomorphic adenoma
hTERT	human telomerase reverse transcriptase
PA	pleomorphic adenoma
rPA	recurrent pleomorphic adenoma
SGT	salivary gland tumour
THOR	TERT hypermethylated oncological region
TPM	TERT promoter mutations
