# Supplementary figures and images for: Methylation Status of the Telomerase Reverse Transcriptase Promoter in Parotid Tumours and Adjacent Parotid Gland Tissue: A Pilot Study on the Implications for Recurrence and Development of Malignancy
Source: Curr Oncol. 2025 May 28;32(6):312. doi: 10.3390/curroncol32060312 (PMC12191565; doi:10.3390/curroncol32060312)

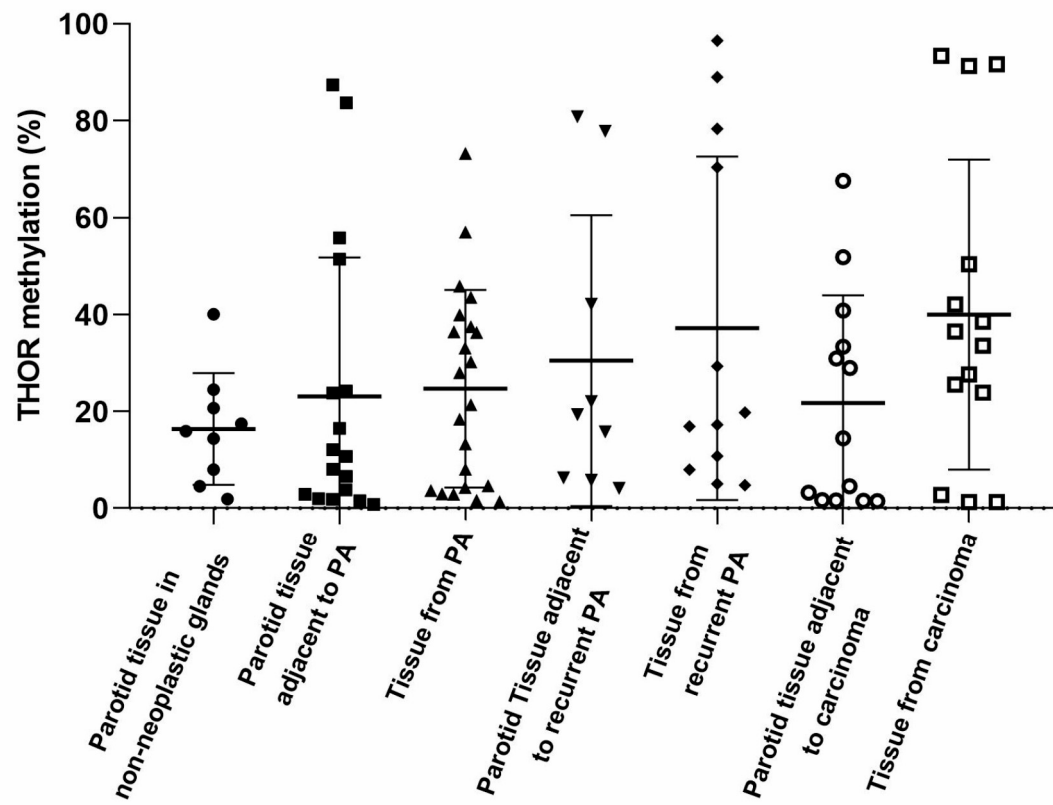

Supplementary Figure S1. THOR methylation in the seven groups.

Supplement: Supplementary file 1 [file curroncol-32-00312-s001.zip › curroncol-3634858-supplementary.pdf]
